# Biofortification and Bioavailability of Rice Grain Zinc as Affected by Different Forms of Foliar Zinc Fertilization

**DOI:** 10.1371/journal.pone.0045428

**Published:** 2012-09-20

**Authors:** Yanyan Wei, M. J. I. Shohag, Xiaoe Yang

**Affiliations:** Ministry of Education (MOE) Key Laboratory of Environmental Remediation and Ecosystem Health, College of Environmental and Resources Science, Zhejiang University, Hangzhou, People’s Republic of China; National Taiwan University, Taiwan

## Abstract

**Background:**

Zinc (Zn) biofortification through foliar Zn application is an attractive strategy to reduce human Zn deficiency. However, little is known about the biofortification efficiency and bioavailability of rice grain from different forms of foliar Zn fertilizers.

**Methodology/Principal Findings:**

Four different Zn forms were applied as a foliar treatment among three rice cultivars under field trial. Zinc bioavailability was assessed by *in vitro* digestion/Caco-2 cell model. Foliar Zn fertilization was an effective agronomic practice to promote grain Zn concentration and Zn bioavailability among three rice cultivars, especially, in case of Zn-amino acid and ZnSO_4_. On average, Zn-amino acid and ZnSO_4_ increased Zn concentration in polished rice up to 24.04% and 22.47%, respectively. On average, Zn-amino acid and ZnSO_4_ increased Zn bioavailability in polished rice up to 68.37% and 64.43%, respectively. The effectiveness of foliar applied Zn-amino acid and ZnSO_4_ were higher than Zn-EDTA and Zn-Citrate on improvement of Zn concentration, and reduction of phytic acid, as a results higher accumulation of bioavailable Zn in polished rice. Moreover, foliar Zn application could maintain grain yield, the protein and minerals (Fe and Ca) quality of the polished rice.

**Conclusions:**

Foliar application of Zn in rice offers a practical and useful approach to improve bioavailable Zn in polished rice. According to current study, Zn-amino acid and ZnSO_4_ are recommended as excellent foliar Zn forms to ongoing agronomic biofortification.

## Introduction

Zinc (Zn) deficiency is a well documented global health problem, affecting nearly half of the world population, particular in developing countries, where high proportion of cereal crops, such as rice and wheat, consumed as a staple food [1], [2]. The reliance on cereal based food induce Zn deficiency related health problem, such as impairments in physical growth, immune system and brain function [3], [4]. Among the cereals, Rice (*Oryza stavia* L.), being one of the leading staple crop for half of the world’s population and, hence, is the main source of Zn to human [5]. Rice, however unfortunately, is a poor source of metabolizable Zn, due to inherently low in Zn content and the bioavailable Zn [6]. Enrichment of rice with high bioavailable Zn is, therefore, suggested as a way to generate major health benefits for a large number of susceptible people.

Zinc biofortification, which aims to enhance Zn concentration as well as bioavailability of rice grain, is considered as the more sustainable and economical solution to address human Zn deficiency [7]. Genetic biofortification and agronomic biofortification are two important agricultural tools to improve rice grain Zn concentration [2], [8]. However, yield factor, interactions between genotype and environment, lack of sufficient genetic diversity in current cultivars for breeding program, consumer resistance and safety of genetically modified crops are the main bottlenecks of genetic biofortification [2], [9], [10], [11]. The traditional and efficient strategy of agronomic biofortification, such as Zn fertilization is, therefore, urgent, essential and rapid solution for improving Zn concentration in rice grain to address the ongoing human Zn deficiency.

Three methods, including soil amendment, seed priming and foliar application, used in Zn fertilizations, have been extensively reviewed [2]. In recent years, a considerable progress has been made on the impact of foliar Zn fertilization on biofortification of Zn in rice grain [12], [13], [14], since it has the advantages of low application rates and avoiding Zn losses through soil fixation [15]. Furthermore, foliar applied Zn caused greater increases in brown rice Zn concentration than soil application [13], [14]. There is evidence in literature demonstrating that foliar applied Zn can be absorbed by leaf epidermis, and remobilized and transferred into the rice grains through the phloem [16] and several members of the Zn-regulated transporters regulate this process [17]. In most of those literatures, the reported data are mostly based on brown rice. As polished rice is the main consumed portion by human, rare information was found on Zn concentration in polished rice after foliar Zn fertilizations. Moreover, time of foliar application and the different forms of foliar Zn fertilizers may differentially influence grain Zn concentration. In recent past, several studies have been conducted to adjust time of foliar Zn application in cereal crops [2], [13], [18]. It is now well established that foliar Zn application after flowering stage (e.g., at early milk plus dough stages) more distinctly increase the grain Zn concentration [13]. On the other hand, different Zn fertilizers such as inorganic and organic Zn salts play a fundamental role in the way in nutrient transport from leave to the grain [19]. Unfortunately, studies evaluating the effectiveness of foliar application of different Zn forms on rice grain Zn accumulation are still rare.

The metabolizable Zn from biofortified crop grain not only depends on net Zn concentration, but also a large extent on the bioavailability of Zn. Zinc bioavailability defined as the proportion of the total amount of Zn that is potentially absorbable in a metabolically active form [20]. Phytic acid, the naturally occurring anti-nutrient presents in the seed, reduces the bioavailability of Zn, because of its ability to form complex with Zn, and inhibits Zn solubility, digestibility and absorption in human body [21]. Although, it is assume that foliar Zn fertilization improved Zn bioavailability, but till now there are rare studies on the Zn bioavailability of rice grain deserved from different forms of foliar Zn application [2]. Hence, an *in vivo* approach to assess the potential benefits of different forms of foliar Zn application on grain Zn bioavailability is required.

Ideally, Zn bioavailability in crop grains should be evaluated through *in vivo* human study. However, complexity to perform large-scale screening of sample and cost limit their applicability [22]. *In vitro* digestion/Caco-2 cell model has been proposed as an alternative to *in vivo* method for estimating mineral bioavailability in diets. In recent years, *in vitro* digestion/Caco-2 cell culture model is being utilized for absorption studies involving Zn. This *in vitro* model is currently considered as the best approach, in term of cost and time, to investigate the bioavailability of different food components as a prelude to *in vivo* study [23]. The present study used this model to assess the bioavailability of Zn from polished rice grain fortified with different forms of foliar Zn fertilization.

Viewing the above circumstances, the current study were aimed: (i) to assess the effect of different forms of foliar Zn fertilizer on Zn concentration in brown rice and polished rice, (ii) to assess the effect of different forms of foliar Zn fertilizer on Zn bioavailability in polished rice. The findings of the current study were used to design experiment to identify some useful foliar Zn fertilizer for increasing the level of bioavailable Zn in rice grain.

## Materials and Methods

### Field Experiment and Sampling

#### Field location

Experimental site was Longyou, Zhejiang province (29° 02′ N, 119° 11′ E), China. The climate of the experimental site is subtropical humid. The soil type of experimental field was periodical water logged paddy soil. Before the start of experiments, puddle layer (0–15 cm top soil) soil samples were taken from four random spots of the field and analyzed for various physico-chemical properties (Table1).

**Table 1:** Selected physical and chemical properties of the soils.

Characteristics	Value
**pH (H_2_O, 20 °C)**	5.8
**Total N (g kg^−1^)**	1.36
**Organic matter (g kg^−1^)**	13.70
**Olsen P (mg kg^−1^)**	42.6
**CaCO_3_ (%)**	1.98
**NH_4_OAC-exchangeable K (mg kg^−1^)**	90.35
**DTPA-extractable Zn (mg kg^−1^)**	3.84
**DTPA-extractable Fe (mg kg^−1^)**	198.45

#### Experimental design and treatment

Experimental design was a split plot with four replications. Foliar Zn fertilization treatments were treated as main plot and rice cultivars as sub-plot. Foliar Zn fertilization treatments comprised of four different forms of Zn fertilizer: (i) ZnNa_2_EDTA (Zn-EDTA), Zn-EDTA was the common Zn fertilizer which contain 9% Zn (ii) Zn-Citrate, in which Zn content was 10% (iii) ZnSO_4_·7H_2_O (ZnSO_4_), common Zn fertilizer, in which Zn content was 36% (iv) Zn-amino acids (Zn-AA), Zn-AA contains Zn as ZnSO_4_ (10%) and amino acid (25%) and, (v) Control, sprayed with distilled water. Three rice (*Oryza sativa* L.) varieties differ in their grain Zn concentration namely Hai7, Bing91185 and Biyuzaonuo were selected according to our previous study [24]. Thus, there were 60 plots with each 4 m^2^ (2×2 m).

Thirty days old seedlings of each cultivar were transplanted to the plot. Before transplanting, the standard recommended dose of NPK fertilizer was applied to all plots at rates of 187.5 kg N ha**^−^** ^1^ (70% applied as basal dose and 30% as topdressing at panicle initiation stage), 70 kg P_2_O_5_ ha**^−^** ^1^ and 93 kg K_2_O ha**^−^** ^1^. Water management was the same as conventional rice farming practice. The foliar Zn was applied three times, one time at panicle initiation stage, two times at 7 days after flowering stage. Spray was applied after sunset. During spray, soil surface was covered to minimize the contamination of soil with foliar applied Zn. The concentration of Zn fertilizer was 0.2%. The amount of foliar Zn applied was equivalent to 2.5 kg Zn ha^−1^. All foliar sprays contained 0.01% (v/v) Tween80 as a surfactant.

#### Rice sample preparation

Plants were harvested from the center of each plot at maturity and were manually threshed to separate grains. Rice grains were air dried; the brown rice was prepared by removing the husk using a laboratory de-husker (JLGJ4.5, Taizhou Cereal and Oil Instrument Co. Ltd., Zhejiang, China), the polished rice was prepared by polishing the bran by a laboratory polishing machine (JNMJ3, Taizhou Cereal and Oil Instrument Co. Ltd., Zhejiang, China). The rice samples were powdered to make flour by using a ball mill (Retsch, MM-301, Germany), then put in the plastic bag and keep at −20°C until analysis. A part of rice was cooked for 15 min with 1∶2 rice/deionized water (w/v). The cooked rice samples were then homogenized in a polytron homogenizer and then the homogenates were frozen and lyophilized before testing via the *in vitro* digestion/Caco-2 cell model.

### Chemical Analysis

#### Mineral concentration determination

The ground rice samples (0.3 g) of each treatment were placed in to PTFE digestion tube and, digested with nitric acid (2 mL) and hydrogen peroxide (0.5 mL). After cooling, the digestion solution was transferred to a 25 mL volumetric flask, made up the volume with deionized water. The concentrations of Zn, iron (Fe), calcium (Ca) in sample were determined by inductively coupled plasma mass spectrometry (ICP-MS, Agilent 7500a, Agilent Technologies, CA, USA) following our previously described method [16].

#### Phytic acid determination

Phytic acid from the rice samples was determined by the method described by Dai et al., [25]. Briefly, 0.5 g of rice flour was extracted with 10 mL of 0.2 M HCl for 2 h by a rotary shaker and then centrifuged at 10000 *g* for 10 min. The clear supernatant was collected, and 2 mL of 0.2% FeCl_3_ was added to 2.5 mL of supernatant. The resulting solution was mixed thoroughly, heated in a boiling water bath for 30 min, cooled in room temperature, and centrifuged at 10000 *g* for 15 min. Then supernatant was discarded and the residue in the tube washed three times with 5 mL of deionized water. The tube was then centrifuged again at 10000 *g* for 10 min after adding 3 mL of 1.5 M NaOH to it. The supernatant was discarded again, and 3 mL of 0.5 M HCl was added to the tube to dissolve the residue. Finally, deionized water was added to the solution made up to the volume of 10 mL. The Fe concentration in the solution was measured by ICP-MS (Agilent 7500a, Agilent Technologies, CA, USA). The phytic acid content was calculated by multiplying Fe content by the factor 4.2.

#### Protein content determination

Rice samples were analyzed for protein content by determination of total nitrogen. The Kjeldahl method was used to determine total nitrogen. Rice samples (0.5 g) were digested by 20 mL H_2_SO_4_, and then distilled in KjelFlex K-360 (Buchi, Flawil, Switzerland) with 40% (w/v) NaOH and 2% (w/v) boric acid (methyl red and bromcresol green used as an indicator solution), then titrated with 0.02 mM H_2_SO_4_. The protein content was subsequently calculated by multiplying nitrogen content by a conversion factor of 5.95 [26].

### Zinc Bioavailability Assay

#### *In vitro* digestion of rice sample

The *in vitro* digestion method according to our previously described method [27]. Briefly, 5 g of cooked rice powder was mixed with 15 mL of saline buffer (140 mM NaCl, 5 mM KCl), and pH was adjusted to 2 with 6 M HCl. Then, the sample was mixed with 0.5 mL of pepsin solution (0.2 g pepsin in 5 mL of 0.1 M HCl) and incubated on a shaking water bath for 2 h at 37°C. After 2 h of gastric digestion, the pH of digest was adjusted to 5.0. The intestinal phase of digestion was then initiated with the addition of 2.5 mL of pancreatin-bile solution (0.45 g of bile salts and 0.075 g of pancreatin in 37.5 mL of 0.1 M NaHCO_3_), and the digest samples were then incubated in a shaking water bath for 2 h. To stop the intestinal digestion, the digest sample was cooled in ice for 10 min, and then the pH was adjusted to 7.2 by adding 0.5 M NaOH. The obtained digests were heated at 100°C for 4 min to inhibit the proteases. The gastrointestinal digest were centrifuged 3500 *g* for 1 h at 4°C. Prior to addition of rice soluble fraction to the cells, glucose (5 mM) and HEPES (50 mM) were added in order to make it similar to culture media. Deionized water was used to adjust the osmolarity to 310±10 mOsm kg**^−^** ^1^ (Freezing point osmometer, Osmomat 030, Berlin, Germany). The supernatants (soluble fraction) were analyzed for Zn content and used in cell uptake assays.

#### Cell culture

The Caco-2 human intestinal cell line was purchased from Institute of Biochemistry and Cell Biology (SIBS, CAS, Shanghai, China) were used in experiments at passage 25–37. The cells were grown in 75 cm^2^ flasks (Corning Inc., NY, USA) and maintained in high-glucose (4.5 g L**^−^** ^1^) Dulbecco’s modified minimal essential medium (GIBCO, Grand Island, NY, USA ), supplemented with 10% (v/v) heat-inactivated fetal bovine serum (GIBCO, Grand Island, NY, USA), 4 mM L-glutamine, 1% (v/v) non-essential amino acids and 1% (v/v) antibiotic solution (GIBCO, Grand Island, NY, USA). The cells were incubated in a cell culture incubator (Heraeus, BB15, Germany) set at 37°C, 5% CO_2_ and 95% atmospheric air at constant humidity. After reaching 80% confluence, the cells were digested by using 0.25% trypsin (GIBCO, Grand Island, NY, USA), then cells were seeded at a density of 50000 cells cm**^−^** ^2^ in 1.5 mL of complete DMEM in polyester membrane chamber inserts (24 mm diameter, 0.4 µm pore size; Costar Corp. NY, USA), the basal compartment contained 2.5 mL of complete Dulbecco’s modified minimal essential medium, and the medium was changed every 2 days. Zinc bioavailability experiments were carried out 21 days after initial seeding. Subsequently, the integrity of Caco-2 cells monolayer was assessed and validated by transepithelial electrical resistance (TEER) using a Millicell-ERS meter (Millipore Corporation, Bedford, MA, USA). The method of TEER was according to the manufacturer’s protocols. Only those filters that had TEER values >250 Ω cm^2^ at the beginning and the end of the experiment were included. The monolayer used in this study exhibited adequate TEER values 500–680 Ω cm^2^.

#### Zinc uptake experiment in Caco-2 cell

Prior to the Zn bioavailability experiment, the growth medium was removed from each culture well, and the cell layer was washed three times with Ca^2+^ and Mg^2+^ free HBSS at 37°C at pH 7.4. Then, 2.5 mL of transport solution (130 mM NaCl, 10 mM KCl, 1 mM MgSO_4_, 5 mM glucose and 50 mM HEPES, pH 7.4) was added to the bottom chamber, and 1.5 mL of rice soluble fraction was added to the apical chamber. Cell cultures were incubated at 37°C under 5% CO_2_, with relative humidity 95% for 2 h. After incubation, basolateral compartrnents were collected for determine of Zn transported across the monolayer. The cell monolayer was washed twice with ice cold HBSS at pH 7.4 to remove nonspecifically bound mineral and residual medium. The cell on filters were lysed by the addition of 1 mL of deionized water in the well, and then harvested. Cell viability after 2 h of exposure to the uptake solution was assessed by trypan blue exclusion and typically 85–95%.

The concentrations of Zn in cell retention (Zn fraction in the cell monolayer) and the solution of transport (Zn collected from basolateral compartment) were assessed by ICP-MS (Agilent 7500a, Agilent Technologies, CA, USA). Zinc uptake in Caco-2 cell model was calculated as the Zn content in cell retention plus the Zn content in transport. Solubility percentages were calculated by using following equation: solubility% = soluble fraction (µg of Zn g**^−^** ^1^ sample)× 100/C, where C = total Zn content of sample; The following equation was used for Zn retention percentage: Zn retention% = Zn retention (µg well**^−^** ^1^) ×100/C, where C = mineral soluble added (µg); The following equation was used for Zn transport percentage: Zn transport% = Zn transport (µg well**^−^** ^1^) × 100/C, where C = mineral soluble added (µg); The following equation was used for Zn uptake percentage: Zn uptake% =  (retention+ transport µg well**^−^** ^1^) × 100/C, where C = mineral soluble added (µg). Due to the differences among samples in terms of solubility of Zn after *in vitro* digestion, Zn uptake availability was expressed as Zn uptake efficiency, Zn uptake efficiency% = (% solubility × % uptake)/100. Bioavailable Zn (µg g**^−^** ^1^ polished rice)  = Zn concentration (mg kg**^−^** ^1^) × Zn uptake efficiency%.

### Quality Control of Mineral Analysis

Standard reference material rice flour (SRM 1568a) from National Institute of Standards and Technology (Gaithersburg, MD, USA) was used to check the accuracy of Zn, Fe and Ca analysis. The measured value was 19.7±0.2 mg kg**^−^** ^1^ for Zn, 6.9±0.3 mg kg**^−^** ^1^ for Fe and 114.5±1.2 mg kg**^−^** ^1^ for Ca, which values were in accordance with the certified ranges of 19.4±0.5 mg kg**^−^** ^1^ for Zn, 7.4±0.9 mg kg**^−^** ^1^ for Fe and 118±6 mg kg**^−^** ^1^ for Ca.

### Statistical Analysis

Statistical analysis of the data was performed using SPSS12.0 (SPSS, Inc., Chicago, IL, USA). The data were subjected to a separate analysis of variance (ANOVA) for each cultivar, and Fisher’s least significant difference (LSD) at P<0.05 was used to determine differences between treatment means. The Pearson correlation procedure and linear regression model was used to evaluate the relationship between brown rice and polished rice Zn concentration.

## Results

### Biomass and Grain Yield

Biomass, grain yield, harvest index and thousand seed weight of rice did not different among the four different forms of foliar Zn treatments for all three rice cultivars (Table 2).

**Table 2:** Effect of different forms of foliar Zn fertilization on the biomass, grain yield, harvest index and thousand seed weight of three rice cultivars.

Treatments	Cultivars^a^					
	Hai7	Bing91185	Biyuzaonuo	Hai7	Bing91185	Biyuzaonuo
	**Biomass (t hm^−1^)**			**Grain yield (t hm^−1^)**		
**Control**	19.50 a	18.61 a	18.35 a	7.81 a	7.98 a	7.50 a
**Zn-EDTA**	19.57 a	18.73 a	18.55 a	7.86 a	7.93 a	7.61 a
**Zn-Citrate**	19.79 a	18.66 a	18.43 a	8.00 a	8.03 a	7.53 a
**ZnSO_4_**	19.63 a	18.39 a	18.61 a	7.97 a	7.92 a	7.64 a
**Zn-AA**	19.14 a	18.84 a	18.80 a	8.01 a	8.04 a	7.66 a
**Zn effect** **by f-test** b	NS	NS	NS	NS	NS	NS
	**Harvest index (%)**			**Thousand seed weight (g)**		
**Control**	40.16 a	42.95 a	40.91 a	22.01 a	22.96 a	20.92 a
**Zn-EDTA**	40.20 a	42.37 a	41.05 a	23.05 a	24.13 a	21.85 a
**Zn-Citrate**	40.44 a	43.11 a	40.87 a	22.12 a	24.52 a	22.02 a
**ZnSO_4_**	40.59 a	43.06 a	41.13 a	22.34 a	23.68 a	22.45 a
**Zn-AA**	41.86 a	42.68 a	40.96 a	23.23 a	24.56 a	22.56 a
**Zn effect** **by f-test** b	NS	NS	NS	NS	NS	NS

a Different letters after number in the same column designated significant difference by LSD_P<0.05._

b Significant effects: NS  =  not significant at P>0.05.

### Zinc Concentration in Brown Rice and Polished Rice

**Figure 1:**
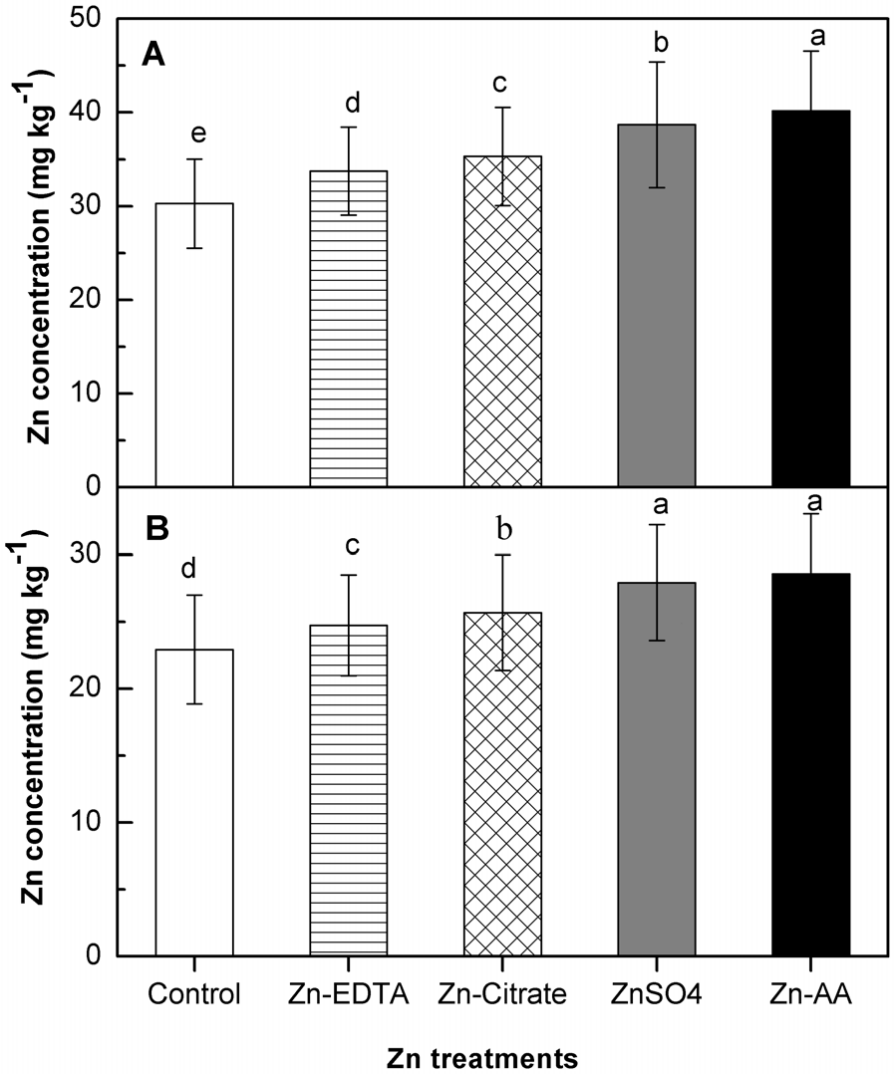
Effect of different forms of foliar Zn fertilization on Zn concentration in rice grain. (A) Zn concentration in brown rice. (B) Zn concentration in polished rice. Error bars indicate standard errors of the means (n = 4). Different letters indicate significant difference among Zn treatments according to LSD test (P<0.05).

Foliar Zn fertilization had significant (P<0.05) impact on Zn concentration in brown rice and polished rice (Fig. 1). Brown rice Zn concentration was significantly increased by foliar Zn fertilizations (Fig. 1A). Regardless of the three cultivars, Zn concentration in brown rice was increased from 30.28 mg kg**^−^** ^1^ in the control, to 33.75 mg kg**^−^** ^1^ by foliar Zn-EDTA application, to 35.07 mg kg**^−^** ^1^ by foliar Zn-Citrate application, to 38.45 mg kg**^−^** ^1^ by foliar ZnSO_4_ application, to 39.84 mg kg**^−^** ^1^ by foliar Zn-AA application, these represented increases of 11.46%, 15.81%, 27.26% and 31.58%, respectively. Zn concentration in polished rice was significantly increased by foliar Zn fertilizations (Fig. 1B). Regardless of the three cultivars, Zn concentration in polished rice was increased from 22.92 mg kg^−1^ in the control, to 25.26 mg kg^−1^ by foliar Zn-EDTA application, to 26.09 mg kg^−1^ by foliar Zn-Citrate application, to 28.08 mg kg^−1^ by foliar ZnSO_4_ application, to 28.67 mg kg^−1^ by foliar Zn-AA application, these represented increases of 10.22%, 13.82%, 22.47% and 24.04%, respectively. Thus, foliar Zn fertilization could increase Zn concentration in brown rice and polished rice depending on Zn form.

**Figure 2:**
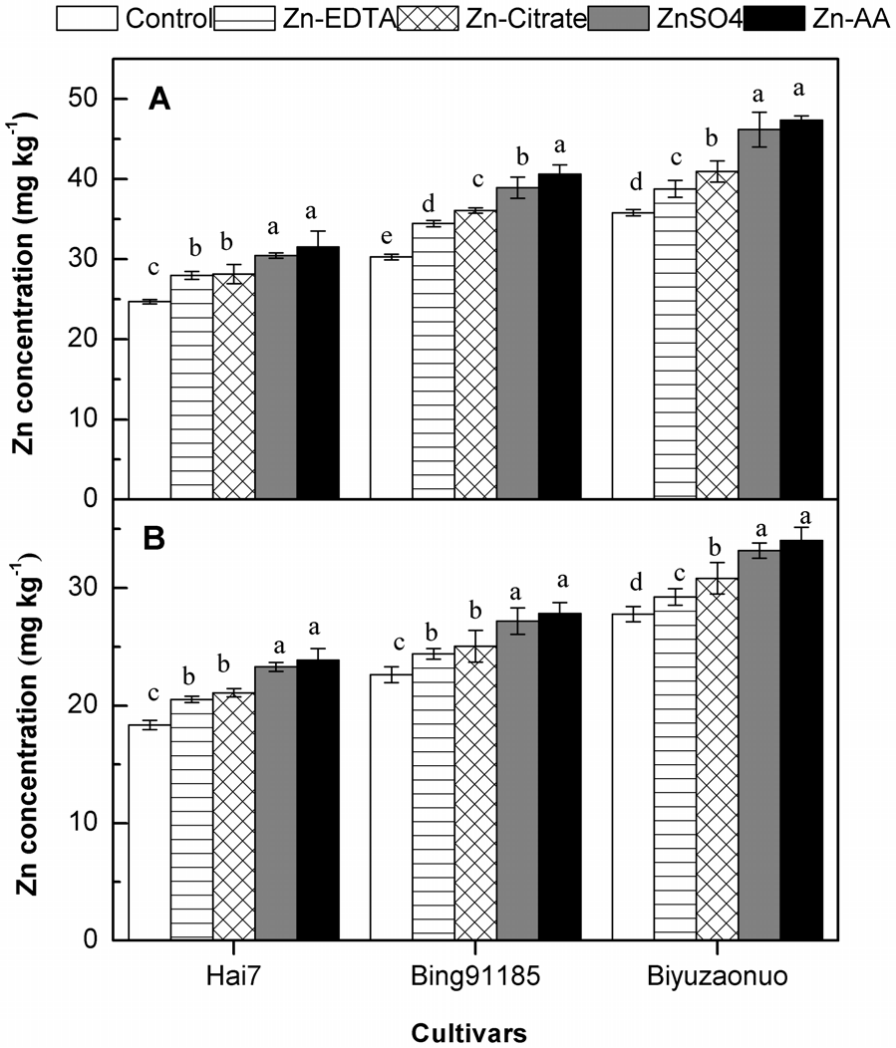
Zn concentration in rice grain among three cultivars. (A) Zn concentration in brown rice. (B) Zn concentration in polished rice. Error bars indicate standard errors of the means (n = 4). Different letters indicate significant difference among Zn treatments according to LSD test (P<0.05).

The concentration of Zn in brown rice and polished rice of all cultivars were significantly increased by different forms of foliar applied Zn (Fig. 2). In control, brown rice Zn concentrations were 24.71, 30.29 and 35.82 mg kg^−1^ in cultivar Hai7, Bing91185 and Biyuzaonuo, respectively. After application of Zn-EDTA through the foliage, Zn concentrations in brown rice were 27.98, 34.47 and 38.79 mg kg^−1^ in cultivar Hai7, Bing91185 and Biyuzaonuo, respectively. After foliar application of Zn-Citrate, Zn concentrations in brown rice were 28.15, 36.08 and 40.96 mg kg^−1^ in cultivar Hai7, Bing91185 and Biyuzaonuo, respectively. After foliar application of ZnSO_4_, Zn concentrations in brown rice were 30.46, 38.94 and 46.20 mg kg^−1^ in cultivar Hai7, Bing91185 and Biyuzaonuo, respectively. After foliar application of Zn-AA, Zn concentrations in brown rice were 31.53, 40.62 and 47.38 mg kg^−1^ in cultivar Hai7, Bing91185 and Biyuzaonuo, respectively (Fig. 2A). Similar trends were found in polished rice (Fig. 2B), the cultivar Biyuzaonuo had the highest Zn concentration, while Hai7 had the lowest Zn concentration in all Zn treatments.

With respect to Zn content, a significant correlation was found between polished rice and brown rice (y = 0.619x +4.232, R^2^ = 0.897, P<0.01).

### Phytic Acid Content in Polished Rice

Foliar Zn fertilization reduced the phytic acid content in the polished rice (Table 3). Regardless of cultivar, phytic acid content in polished rice ranged from 2.25 mg g^−1^ in the control, to 2.09 mg g^−1^ by Zn-EDTA, to 2.06 mg g^−1^ Zn-Citrate, to 1.87 mg g^−1^ by foliar ZnSO_4_ and 1.92 mg g^−1^ by Zn-AA, these showed that, the decreases of 6.97%, 8.42%, 16.91% and 14.57%, respectively.

**Table 3:** Effect of different forms of foliar Zn fertilization on the grain protein, phytic acid, Fe and Ca contents of three rice cultivars.

Treatments	Cultivars^a^					
	Hai7	Bing91185	Biyuzaonuo	Hai7	Bing91185	Biyuzaonuo
	**Protein (%)**			**Phytic acid (mg g** ^−**1**^ **)**		
**Control**	9.06 b	9.32 b	9.98 a	1.78 a	1.64 a	3.32 a
**Zn-EDTA**	9.15 b	9.34 b	9.99 a	1.66 b	1.48 b	3.14 b
**Zn-Citrate**	9.16 b	9.66 ab	9.91 a	1.65 b	1.47 b	3.05 b
**ZnSO_4_**	9.15 b	9.37 b	9.99 a	1.54 c	1.39 c	2.89 c
**Zn-AA**	9.43 a	9.76 a	10.17 a	1.50 c	1.36 c	2.67 d
**Zn effect** **by f-test** b	*	*	NS	***	***	***
	**Fe (mg kg** ^−**1**^ **)**			**Ca (mg kg** ^−**1**^ **)**		
**Control**	2.92 a	3.35 a	5.89 a	92.00 a	110.22 a	146.48 a
**Zn-EDTA**	3.12 a	3.74 a	5.84 a	83.67 a	107.13 a	150.49 a
**Zn-Citrate**	3.10 a	3.81 a	5.73 a	96.38 a	112.01 a	145.90 a
**ZnSO_4_**	2.98 a	3.88 a	5.69 a	91.00 a	108.86 a	146.89 a
**Zn-AA**	2.98 a	3.86 a	5.79 a	94.12 a	107.43 a	143.80 a
**Zn effect** **by f-test** b	NS	NS	NS	NS	NS	NS

a Different letters after number in the same column designated significant difference by LSD_P<0.05._

b Significant effects: NS  =  not significant at P>0.05;

* at P<0.05;

** at P<0.01;

*** at P<0.001.

### Protein, Iron and Calcium Concentration in Polished Rice

Protein content in polished rice showed an increase trend by foliar Zn-AA, but not by other foliar Zn fertilizations (Table 3). Foliar application of Zn-AA had significant impact on grain protein content in cultivar Hai7 and Bing91185, except Biyuzaonuo. Generally, compared to the control, foliar Zn-AA could increase protein content by 1.88–4.79% depending on cultivar. Grain Fe and Ca concentration did not change by different forms of Zn treatments in all three cultivars. Foliar Zn fertilization had little impact on Fe and Ca content (Table 3).

### *In vitro* Zinc Solubility of Polished Rice

The amount of Zn solubilized after *in vitro* digestion is an indicator for bioavailability. Foliar Zn fertilization had significant (P<0.05) effect on Zn solubility (Table 4). Averaged across the cultivars, *in vitro* Zn solubility from polished rice was ranged from 28.48% in the control, to 29.34% by application of Zn-EDTA, 29.41% by application of Zn-Citrate, 31.15% by application of ZnSO_4_ and 30.67% by application of Zn-AA, these represented increase of 2.99%, 3.24%, 9.36% and 7.65%, respectively. Foliar application of Zn-AA and ZnSO_4_ significant improved *in vitro* Zn solubility in all cultivars.

**Table 4:** Effect of different forms of foliar Zn fertilization on the percentages of solubility, retention, transported and uptake efficiency of Zn among three rice cultivars.

Treatments	Cultivars^a^					
	Hai7	Bing91185	Biyuzaonuo	Hai7	Bing91185	Biyuzaonuo
	**Solubility (%)**			**Retention (%)**		
**Control**	29.17 c	30.58 c	25.70 b	14.53 c	14.91 b	14.39 c
**Zn-EDTA**	30.75 c	31.21 bc	26.06 b	14.82 bc	14.96 b	15.04 bc
**Zn-Citrate**	30.90 bc	31.13 bc	26.20 b	14.93 bc	15.78 b	15.11 bc
**ZnSO_4_**	32.68 a	32.24 a	28.54 a	16.68 ab	18.29 a	16.80 ab
**Zn-AA**	31.64 ab	32.62 a	27.73 a	17.54 a	18.80 a	17.53 a
**Zn effect** **by f-test** b	*	*	***	*	***	*
	**Transport (%)**			**Uptake efficiency (%)**		
**Control**	9.35 b	16.09 b	8.99 c	6.95 c	9.48 b	6.01 b
**Zn-EDTA**	9.61 b	16.23 b	9.57 c	7.52 bc	9.73 b	6.39 b
**Zn-Citrate**	11.08 ab	16.15 b	9.96 bc	8.02 b	9.94 b	6.57 b
**ZnSO_4_**	13.27 a	18.43 ab	13.53 a	9.79 a	11.84 a	8.66 a
**Zn-AA**	13.05 a	19.11 a	12.42 ab	9.68 a	12.39 a	8.30 a
**Zn effect** **by f-test** b	**	*	*	***	***	***

a Different letters after number in the same column designated significant difference by LSD_P<0.05._

b Significant effects: NS  =  not significant at P>0.05;

* at P<0.05;

** at P<0.01;

*** at P<0.001.

### Zinc Bioavailability of Polished Rice

**Figure 3:**
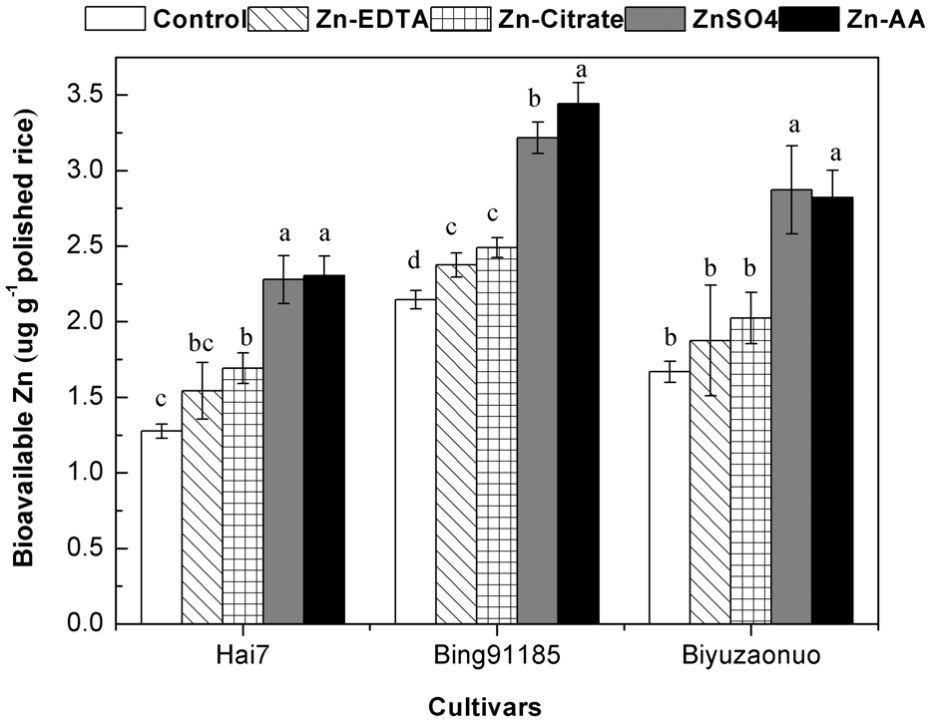
The amount of bioavailable Zn in the polished rice among three cultivars. Error bars indicate standard errors of the means (n = 4). Different letters indicate significant difference among Zn treatments according to LSD test (P<0.05).

The soluble fraction obtained from *in vitro* digestion was used to carry out the retention, transport and uptake experiments in Caco-2 cell (Table 4). Foliar Zn fertilization had significant (P<0.05) impact on the percentages of Zn retention, transport and uptake efficiency in polished rice grain for all cultivars. Generally, foliar application of Zn-EDTA, Zn-Citrate, ZnSO_4_ and Zn-AA could increase the percentages of Zn retention, transport and uptake efficiency in polished rice grain, but only in case of foliar application ZnSO_4_ and Zn-AA could reach significant level in most of the cultivars tested (P<0.05). Regardless of cultivar, compare to the control, after foliar application of ZnSO_4_, the percentages of Zn retention, transport, and uptake efficiency from the polished rice increased by 12.96%, 31.36% and 34.98%, respectively; after foliar application of Zn-AA, the percentages of Zn retention, transport, and uptake efficiency from the polished rice increased by 19.4%, 29.47% and 35.25%, respectively. Regardless of cultivar, the amount of bioavailable Zn in the rice grain has the similar trend as the Zn uptake efficiency (Fig. 3). Regardless of cultivar, compared to the control, amount of bioavailable Zn was increased by 13.85%, 21.96%, 64.43% and 68.37% by Zn-EDTA, Zn-Citrate, ZnSO_4_ and Zn-AA, respectively. Generally, the amount of bioavailable Zn could increased by the foliar Zn fertilizations (Zn-EDTA, Zn-Citrate, ZnSO_4_ and Zn-AA), but only foliar ZnSO_4_ and Zn-AA reach at significant level (P<0.05) in all cultivars tested.

## Discussion

The DTPA extractable Zn (Table 1) in the soil of the current field study was higher than the critical level for rice (0.8 mg kg^−1^) [28], thus the plant was in the sufficient Zn nutritional status. In the Zn sufficient soil, excess foliar application of Zn did not affect the biomass, grain yield, harvest index and thousand seed weight (Table 2). Similar results also found in previous reports [12], [29].

In contrast to grain yield, foliar Zn fertilization significantly (P<0.05) increased the Zn concentration in brown rice (Fig. 1A), consisted with the previous studies [16], [30]. Thus, foliar application Zn as an effective method could boost Zn level in rice grain. Here, in this study, particular attention should be given in the Zn concentration of polished rice, as this is the predominant fraction consumed by human. In current study, we found that regardless of cultivar, although polishing process decrease substantial amount Zn from mature grain, the polished rice obtained from foliar Zn applications was still contained 10.22–24.04% more Zn than those of control (Fig. 1B), and a significant positively correlation in Zn concentration between polished rice and brown rice was also found (R^2^ = 0.897, P<0.01), indicating that Zn concentration in polished rice might be improved by increasing the Zn concentration in brown rice, consisted with previous studies [18], [29], suggesting that excess foliar applied Zn could penetrate into the inner layers of rice endosperm. However, the mechanism of absorbed Zn from aleurone layer into rice endosperm still not clearly, some recent studies reported that nicotianamine and deoxymugineic acid play important role in this process [17], [31].

Furthermore, the effectiveness of foliar Zn fertilization on Zn concentration of brown rice and polished rice varied with the forms of Zn fertilizer (Fig. 1). Among the forms tested for foliar application, foliar Zn-AA and ZnSO_4_ were more effective than Zn-EDTA and Zn-Citrate in improving the Zn concentration in brown rice and polished rice, the results agreed with the previous study [32]. The reasons might be due to the different capacity of leaf penetration of different forms of foliar applied Zn fertilizer [19]. Foliar fertilizer with low molecular weight like Zn-AA and ZnSO_4_ might be easily penetrate into the leaves than those of Zn-EDTA and Zn-Citrate with the high molecular weight, as a result, the more plant-available Zn from foliage might be transported and accumulated in rice grain [19], [33]. The response of grain Zn concentration to foliar Zn fertilization was cultivar dependent (Fig. 2). The results consisted with the previous studies reported rice genotypes differ greatly in their response to foliar applied Zn to increase grain Zn concentration [14], [16]. Thus, impact of foliar Zn application on grain Zn can be maximized by selecting genotypes with higher ability in leaf absorption and seed deposition of foliar applied Zn with low molecular weight.

Although the amount of grain Zn is important for Zn bioavailability, information about changes of anti-nutrients and other nutrients in rice grain especially polished rice during foliar Zn applications is crucial, because it related with Zn bioavailability and nutritional quality of rice, which impact on global human health. Phytic acid has long been known as a form of stored phosphorus in seeds, which was considered as an inhibitor of Zn bioavailability in rice grain [34]. In the current study, it was documented that foliar Zn applications could significantly reduced phytic acid content in polished rice, especially in case of Zn-AA and ZnSO_4_ applications (Table 3), consisted with the previous studies [29], [35]. Possible explanation was that foliar Zn application could inhibit the conversion of inorganic phosphorus to phytic acid in rice grain [2], [35]. Iron and Ca in rice grain are also important minerals because they are frequently deficient in human. In current study, the concentrations of Fe and Ca were varied with cultivar, but not by the foliar Zn application (Table 3). Foliar Zn fertilization had significant effect on grain protein content except the cultivar Biyuzaonuo, however, grain protein content showed increasing tread by foliar application of Zn-AA in all cultivars (Table 3), the results consisted with previous studies [12], [36]. Consequently, above results indicated that foliar Zn application could reduce the phytic acid content, while could maintain the Fe, Ca and protein content in polished rice grain.

*In vivo* situation, Zn needs to be in soluble before it can be taken up by the enterocytes. In current study, we also determined the soluble Zn in polished rice obtained from the different foliar Zn treatments by *in vitro* digestion. The results showed that regardless of cultivar, the solubility of Zn in polished rice from foliar Zn application was higher than control, especially in the case of foliar applications of Zn-AA and ZnSO_4_ (Table 4). To the best of our knowledge, no literature data on regarding foliar Zn fertilizers on the *in vitro* Zn solubility in rice grain are yet available. One possible reason was the *in vitro* Zn solubility in grain was increased by the reduction of phytic acid in grain [2], [37]. The solubility method involves a simulation of gastrointestinal digestion followed by a measurement of soluble Zn in the digest and thus covers only the first phase of the overall Zn absorption process. The soluble Zn fraction obtained form *in vitro* digestion was used to carry out retention, transport and uptake experiment in Caco-2 cell model, which offer a more physiological tool for screening Zn bioavailability in food matrices [27], [37], [38], [39]. In the current study, the percentage of Zn uptake efficiency of polished rice ranged from 6.01% to 12.33%, falling within the previous reported Zn uptake efficiency in Caoc-2 cell model from cereal foods (ranging from 4.1% to 48.1%) [37]. Compared to the control, foliar Zn-AA and ZnSO_4_ could significantly increase the percentages of Zn retention, transport and uptake efficiency of polished rice in most of the cultivars tested (Table 4). Regardless of cultivar, the amount of bioavailable Zn has the similar trend as Zn uptake efficiency (Fig. 3), showing the foliar Zn could improve the amount of bioavailable Zn in the polished rice, especially foliar Zn-AA and ZnSO_4_ could increased by 68.37% and 64.43%, respectively. The results indicated that foliar Zn application could improve the bioavailability of Zn from polished rice but depended on the forms of Zn application. Till now, few literatures on the Zn bioavailability of biofortified polished rice obtained from the foliar Zn fertilization are available. The possible explanation of our results might be foliar Zn-AA and ZnSO_4_ have the higher efficiency to decrease the phytic acid, and improve the total amount of Zn than Zn-EDTA and Zn-Citrate, as a result, increase the amount of bioavailable Zn in the polished rice grain. In addition, in the current study, we also observed that the polished rice of cultivar Biyuzaonuo contained the highest amount of Zn, interestingly, it was not contained the highest amount of bioavailable Zn, this is might be due to presence of significant amount of phytic acid or other anti-nutrients in this cultivar [40]. Thus, it is suggested that not only the net grain Zn concentration but also the bioavailability of grain Zn should be considered in ongoing breeding program.

In conclusion, foliar Zn fertilization was an effective agronomic practice to promote grain Zn concentration and Zn bioavailability, especially, in case of Zn-AA and ZnSO_4._ On average, Zn-AA and ZnSO_4_ increased Zn concentration in polished rice up to 24.04% and 22.47%, respectively. On average, Zn-AA and ZnSO_4_ increased Zn bioavailability in polished rice up to 68.37% and 64.43%, respectively. The effectiveness of foliar applied Zn-AA and ZnSO_4_ were higher than Zn-EDTA and Zn-Citrate to improve the Zn concentration, and reduction of phytic acid, as a results higher accumulation of bioavailable Zn in polished rice. Moreover, foliar Zn application could maintain the protein and minerals (Fe and Ca) quality of the polished rice. Therefore, it’s believed that foliar application of suitable Zn form is a feasible approach to improve the bioavailable Zn status in polished rice.
